# Curcumin at Low Doses Potentiates and at High Doses Inhibits ABT-737-Induced Platelet Apoptosis

**DOI:** 10.3390/ijms22105405

**Published:** 2021-05-20

**Authors:** Natalia Rukoyatkina, Valentina Shpakova, Julia Sudnitsyna, Michael Panteleev, Stephanie Makhoul, Stepan Gambaryan, Kerstin Jurk

**Affiliations:** 1 Sechenov Institute of Evolutionary Physiology and Biochemistry, Russian Academy of Sciences, 194223 St. Petersburg, Russia; natalia.rukoyatkina@gmail.com (N.R.); spakovavalentina@gmail.com (V.S.); julia.sudnitsyna@gmail.com (J.S.); or gambaryan@klin-biochem.uni-wuerzburg.de (S.G.); 2 Center for Theoretical Problems of Physicochemical Pharmacology, Russian Academy of Sciences, 109029 Moscow, Russia; mapanteleev@yandex.ru; 3 Center for Thrombosis and Hemostasis (CTH), University Medical Center of the Johannes Gutenberg University of Mainz, 55131 Mainz, Germany; stephaniemakhoul@live.com

**Keywords:** platelets, apoptosis, autophagy, procoagulant activity, thrombin

## Abstract

Curcumin is a natural bioactive component derived from the turmeric plant *Curcuma longa*, which exhibits a range of beneficial activities on human cells. Previously, an inhibitory effect of curcumin on platelets was demonstrated. However, it is unknown whether this inhibitory effect is due to platelet apoptosis or procoagulant platelet formation. In this study, curcumin did not activate caspase 3-dependent apoptosis of human platelets, but rather induced the formation of procoagulant platelets. Interestingly, curcumin at low concentration (5 µM) potentiated, and at high concentration (50 µM) inhibited ABT-737-induced platelet apoptosis, which was accompanied by inhibition of ABT-737-mediated thrombin generation. Platelet viability was not affected by curcumin at low concentration and was reduced by 17% at high concentration. Furthermore, curcumin-induced autophagy in human platelets via increased translocation of LC3I to LC3II, which was associated with activation of adenosine monophosphate (AMP) kinase and inhibition of protein kinase B activity. Because curcumin inhibits P-glycoprotein (P-gp) in cancer cells and contributes to overcoming multidrug resistance, we showed that curcumin similarly inhibited platelet P-gp activity. Our results revealed that the platelet inhibitory effect of curcumin is mediated by complex processes, including procoagulant platelet formation. Thus, curcumin may protect against or enhance caspase-dependent apoptosis in platelets under certain conditions.

## 1. Introduction

Curcumin derived from the turmeric plant *Curcuma longa* is used in Asia as a culinary spice and has been used in traditional medicine for thousands of years. The structure of curcumin, a natural polyphenolic compound with complex biophysical characteristics, which allows interaction with numerous different proteins, could explain its diverse pharmacological effects. By the classification of Baell [1], curcumin belongs to Pan Assay Interference Compounds (PAINS), which exhibit multiple behaviors and could interfere in different assay readouts such as protein reactivity, redox cycling, and metal chelation. In numerous literature beneficial effects of curcumin have been described such as antioxidant [2], immunomodulatory [3], anti-inflammatory [4,5], anticancer [6,7,8], and antithrombotic [9].

Antithrombotic effects of curcumin are mediated by complex reactions with endothelial cells, with the blood coagulation system, and by inhibition of platelet aggregation. Inhibitory effects of curcumin on platelet aggregation induced by several agonists were characterized in several reports [9,10,11]. Prevention of platelet activation and aggregation by curcumin [10,12,13] includes inhibition of cyclooxygenase and lipoxygenase activity, and consequently thromboxane B2 and 12-HETE generation [14,15]. However, prevention of collagen-induced platelet activation and aggregation was independent of cyclooxygenase activity and associated with inhibition of Syk kinase and of the subsequent activation of PLCγ2.

Curcumin alone or in combination with anticancer drugs is often used for the treatment of different types of cancer [16,17]. In cellular models [18,19,20] and in vivo studies [21,22], including several clinical trials [23,24], the beneficial effects of curcumin on cancer development were described. Clinical applications of curcumin are restricted because of low bioavailability, poor solubility, low intestinal absorption, and rapid metabolism [25]. Therefore, curcumin is currently used as an adjuvant to anticancer compounds formulated in nanoparticles [25,26,27]. Curcumin induces apoptosis through different pathways, including activation of caspase 3 in several cancer cell lines [28,29,30].

Inhibition of platelet activation by curcumin is well documented [9,31]. However, it is still unknown whether curcumin induces apoptosis or autophagy, the formation of procoagulant platelets, or whether it influences platelet apoptosis induced by the precursor of the anticancer drug ABT-737.

In this study, we showed that curcumin induces procoagulant platelet formation that results in strong surface exposure of anionic phospholipids such as phosphatidylserine (PS), loss of mitochondrial membrane potential, and microparticle formation. Curcumin inhibited P-gp, strongly activated AMP kinase (AMPK), inhibited even basal protein kinase B (PKB) activity, and induced autophagy expressed by conversion of LC3I to LC3II. Curcumin itself did not activate caspase 3-dependent apoptosis; however, curcumin at low doses potentiated, and at high doses inhibited ABT-737-induced platelet apoptosis.

## 2. Results

### 2.1. Curcumin Inhibits Thrombin-Induced Platelet Activation but Does Not Stimulate Caspase 3-Dependent Apoptosis

**Figure 1:**
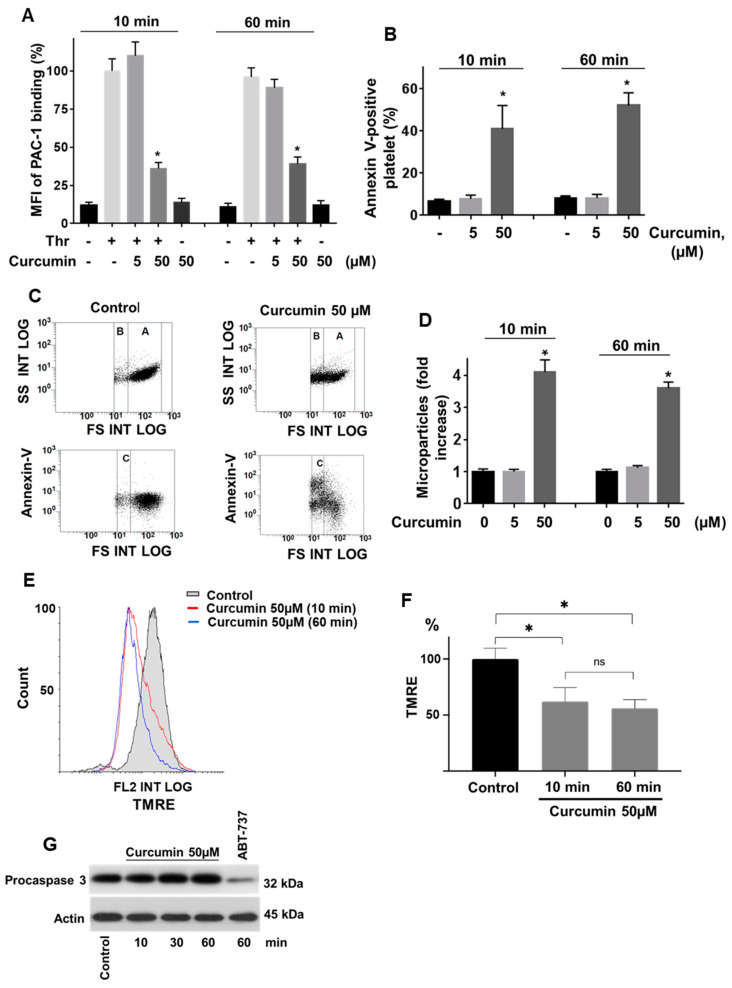
Curcumin inhibits thrombin-induced platelet αIIbβ3 integrin activation and does not stimulate caspase 3-dependent apoptosis. (**A**) Flow cytometric analysis of αIIbβ3 integrin activation (PAC-1-FITC binding), (**B**) PS surface exposure (annexin-V-PE binding), (**C**,**D**) microparticle formation, (**E**,**F**) mitochondrial membrane potential changes (TMRE fluorescence), and (**G**) Western blot of caspase 3 activation. Washed platelets (WP 1 × 10^8^ /mL in A-F and 3 × 10^8^/mL in G) were incubated with the indicated concentrations of curcumin for 10 and 60 min. (**A**) Thrombin (0.01 U/mL) was added for 2 min, followed by PAC-1-FITC antibody (1:10 dilution) for 10 min, and the reaction was stopped by dilution (10 volumes) with PBS. (**B**) WP were incubated with the indicated concentrations/time of curcumin, then annexin-V-PE (dilution 1:10) was added for an additional 10 min, and the reaction was stopped by dilution (10 volumes) with the annexin-V-binding solution. (**C**) Representative (from four independent experiments) dot plot of microparticle formation (upper panel), and annexin-V-PE positive platelets and microparticles (lower panel). Annexin-V-PE was analyzed as shown in B. (**D**) Quantification of platelet microparticle formation. Microparticles were quantified as CD42a positive events in the gate B. (**E**,**F**) WP were incubated with curcumin (50 µM, 10 and 60 min), TMRE dye (dilution 1:10) was added for an additional 10 min, and samples were diluted (10 volumes) with PBS. (**G**) WP were incubated with the indicated concentrations/time of curcumin and processed for Western blotting with caspase 3 antibody (1:1000). ABT-737 was used as positive control and actin blot served as a loading control. All data are presented as means ± SD. Data in A are presented as % of MFI (thrombin sample represents 100%, one-way ANOVA, Levene’s test *p* > 0.05 followed by Tukey’s HSD test, * *p* = 0.0001 compared to controls, *n* = 5). In B, as % of annexin-V positive platelets (one-way ANOVA, Levene’s test *p* > 0.05 followed by Tukey’s HSD test, * *p* = 0.0001 compared to controls, *n* = 5). In D, as fold increase of microparticles (control taken as 1), one-way ANOVA, Levene’s test *p* < 0.05 followed by Tamhane’s T2 test, * *p* = 0.0001 compared to controls, *n* = 5). In F, as % of TMRE fluorescence intensity change (control represents 100%, one-way ANOVA, Levene’s test *p* > 0.05 followed by Tukey’s HSD test, * *p* < 0.05, n.s.—not significant, *n* = 6).

Curcumin, by activation of several apoptotic pathways, can induce apoptosis in cancer cells [19,28,32]. Apoptosis significantly prevents platelet activation [33,34]. Therefore, we tested whether curcumin-mediated platelet inhibition results in activation of apoptotic pathways in platelets. Curcumin itself, even at a high concentration (50 µM), had no effect on platelet activation after 10 and 60 min of incubation (Figure S1). In contrast, 50 µM of curcumin significantly inhibited thrombin-induced integrin αIIbβ3 activation (Figure 1A) and thrombin-induced intracellular Ca^2+^-mobilization (Figure S2). Platelets incubated with 50 µM curcumin showed substantial autofluorescence in the flow cytometric FL1 channel (data not shown). To quantify the specific Fluo-3 signal, representing intracellular Ca^2+^-mobilization, the autofluorescent signal of 50 µM curcumin samples was subtracted from Fluo-3 signals prior to and after the addition of thrombin, respectively (Figure S2B,C). Platelet inhibition was strongly associated with the increase of annexin-V-binding (Figure 1B), microparticle formation (Figure 1C,D), and decrease of mitochondrial membrane potential (Figure 1E,F).

All these events are reliable hallmarks of apoptotic/necrotic cells or procoagulant platelets. Therefore, we tested whether curcumin activates caspase 3-dependent apoptosis in platelets or reduces platelet viability. Incubation of platelets with 50 µM curcumin up to 60 min did not activate caspase 3. The BH3 mimetic ABT-737, a well-known inducer of platelet apoptosis [33,34], was used as a positive control (Figure 1G). For the evaluation of platelet viability, we used a well-established test based on the dye calcein-AM, a fluorogenic substrate of intracellular esterases [35,36]. Platelet viability was not affected by a low concentration of curcumin (5 µM) and was reduced by 17% at 50 µM of curcumin (Figure S3). These results indicate that curcumin-mediated inhibition of platelet activation in response to thrombin is caspase 3-independent and associated with the formation of procoagulant platelets and with a slight reduction of platelet viability.

### 2.2. Curcumin Inhibits P-gp Function in Platelets

**Figure 2:**
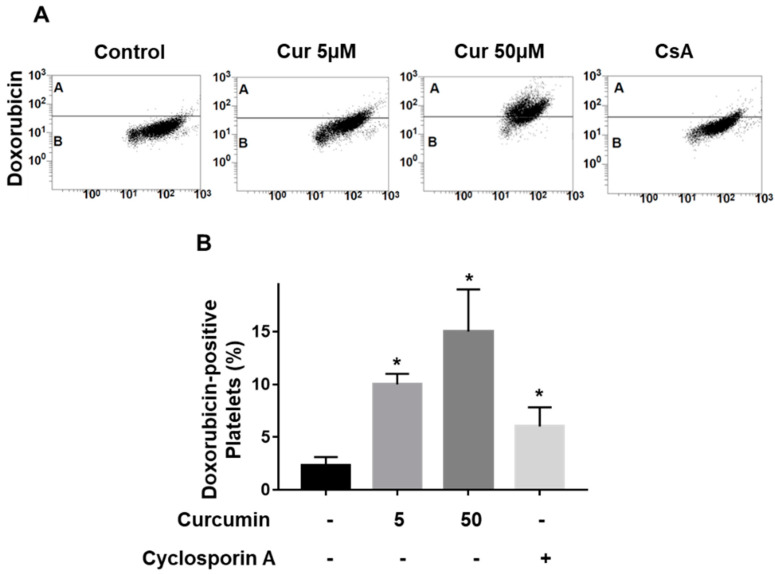
Curcumin increases doxorubicin accumulation in platelets. Washed platelets (1 × 10^8^ /mL) were incubated with the indicated concentrations of curcumin alone or in combination with doxorubicin (20 µM) for 60 min in the absence (control) and analyzed by flow cytometry for doxorubicin fluorescence. P-gp inhibitor cyclosporin A was used as a positive control. (**A**) Representative dot plots, (**B**) quantification of six independent experiments (curcumin autofluorescence was subtracted from 50 µM curcumin samples). Data are presented as means ± SD (One-way ANOVA, Levene’s test *p* < 0.05 followed by Tamhane’s T2 test, * *p* = 0.01 compared to control, *n* = 4).

In cancer cells curcumin inhibited P-gp [7]. P-gp is expressed in the platelet plasma membrane [37]; however, the question of whether curcumin could inhibit P-gp function in platelets is still open. For analysis of P-gp function in platelets we used doxorubicin, which intracellular accumulation reflects the activity of this protein [38]. Doxorubicin at high concentrations (starting from 50 µM) induced mitochondria-mediated intrinsic platelet apoptosis [39]. Therefore, we used a low concentration (20 µM), which had no effects on platelets (data not shown). Platelets incubated with curcumin showed some autofluorescence in the FL1 channel. Therefore, we first measured the signal from curcumin alone and for quantification these data were subtracted from samples of curcumin plus doxorubicin (Figure 2).

The addition of curcumin (5 and 50 µM) significantly increased doxorubicin accumulation. As a positive control for P-gp inhibition by curcumin we used cyclosporine A, one of the well-established inhibitors of this protein [40] (Figure 2). These data indicate that curcumin, similarly to cancer cells, inhibits P-gp activity in platelets.

### 2.3. Curcumin-Induced Autophagy by Conversion of LC3I to LC3II Associated with Stimulation of AMPK and Inhibition of PKB Activity in Platelets

**Figure 3:**
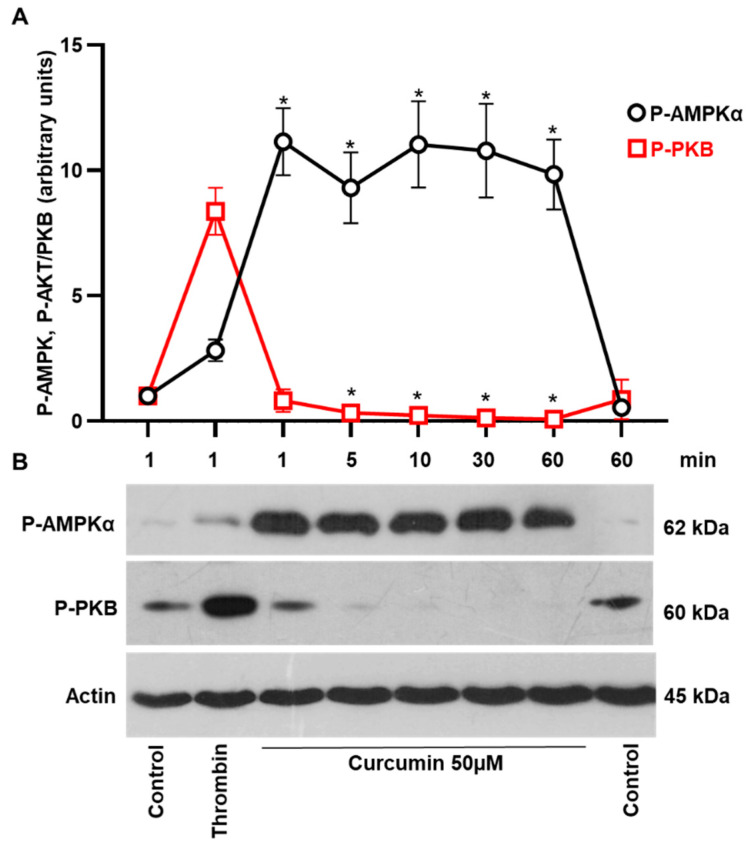
Curcumin stimulates AMPK and inhibits PKB activity in platelets. Washed platelets (3 × 10^8^ cells/mL) were incubated with curcumin (50 µM) for the indicated time and analyzed by Western blotting for total actin, AMPK, and PKB phosphorylation. Thrombin (0.01 U/mL, 1 min) was used as a positive control for PKB and AMPK phosphorylation. (**A**) Quantitative analysis of PKB and AMPK phosphorylation. Immunoblots were scanned and quantified by the Image J program. The intensity of the P-AMPK and P-PKB signal was normalized to the actin signal. For each sample, this ratio is relatively expressed to the ratio for control, which is presented as 1. Data are presented as means ± SD. One-way ANOVA, Levene’s test *p* > 0.05 followed by Tukey’s HSD test for P-AMPK and Levene’s test *p* < 0.05 followed by Tamhane’s T2 test for P-PKB. For P-AMPK * as appeared in the figure *p* = 0.0001; 0.0001; 0.0001; 0.0001; 0.0001, *n* = 4. For P-PKB * *p* = 0.049; 0.012; 0.02; 0.001 (*n* = 5), compared to controls taken as 1 in both cases. (**B**) Representative Western blots of PKA and AMPK phosphorylation.

Our results (Figure 1) showed that curcumin induces the formation of procoagulant platelet without caspase 3 activation. Because curcumin is known to induce cellular autophagy in several cancer cells [41,42,43], we next tested whether this compound initiates autophagy in platelets. Activation of AMPK and inhibition of phosphoinositide 3-kinase (PI3K)/PKB pathways are involved in the induction of autophagy [44]. Therefore, we evaluated the curcumin-mediated activity of these kinases. Curcumin (50 µM, 5 min incubation, and longer) significantly activated AMPK, assessed by phosphorylation of Thr172, and strongly inhibited even basal PKB phosphorylation at Ser473 (Figure 3).

**Figure 4:**
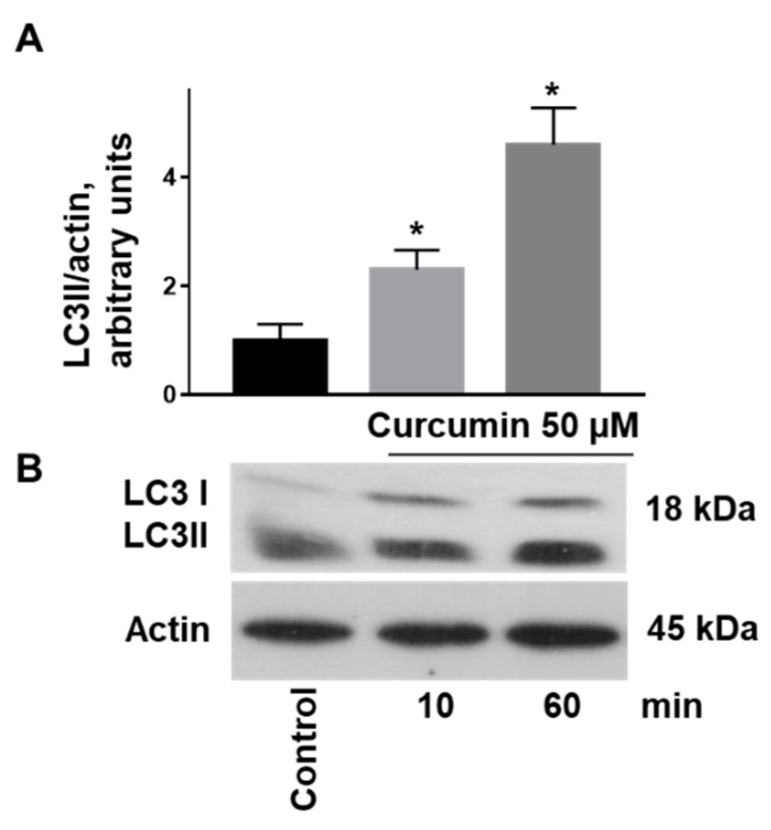
Curcumin induces the conversion of LC3I to LC3II in platelets. Washed platelets (3 × 10^8^ /mL) were incubated with curcumin (50 µM) for the indicated time and analyzed by Western blotting for LC3 translocation (LC3I/LC3II). (**A**) Immunoblots were scanned and the intensity of bands was quantified by the ImageJ program. The intensity of the LC3/II signal was normalized to the actin signal. For each sample, this is relatively expressed to the ratio for the control, which is presented as 1. Data are presented as means ± SD. Data are presented as means ± SD. One-way ANOVA, Levene’s test *p* < 0.05 followed by Tamhane’s T2 test, * as appeared in the figure *p* = 0.002; *p* = 0.019, *n* = 5, compared to controls taken as 1. (**B**) Representative Western blot of LC3 translocation.

Because translocation of LC3 I to LC3 II is one of the established autophagy markers [45], we used this marker for the evaluation of curcumin-induced autophagy in platelets. Curcumin (50 µM, 10 min incubation, and longer) significantly increased LC3 II immunoreactivity, which remained significantly for 60 min of incubation (Figure 4).

### 2.4. Curcumin Had No Effect on ABT-737-Induced Platelet Phosphatidylserine Surface Exposure

**Figure 5:**
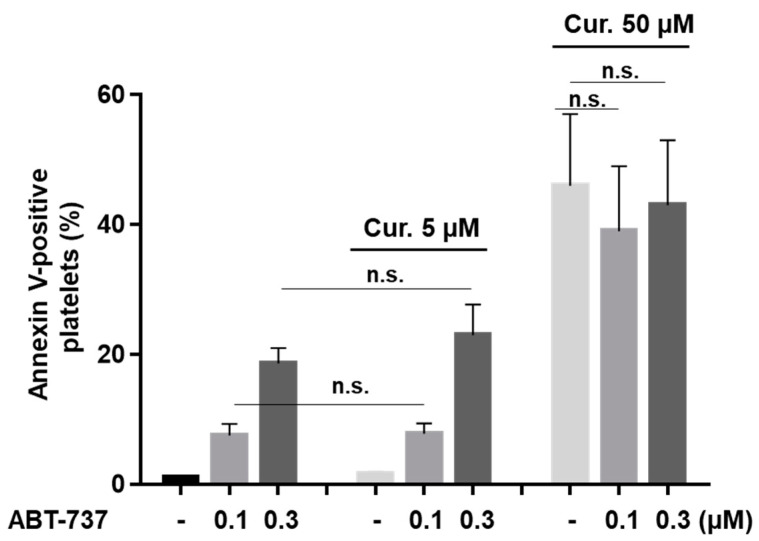
Curcumin had no effect on ABT-737-induced phosphatidylserine surface exposure. Washed platelets (1 × 10^8^ /mL) were incubated with indicated concentrations of ABT-737 in the absence or presence of curcumin (5, 50 µM) and PS-positive platelets (annexin-V-PE binding) were analyzed by flow cytometry, Data are presented as means ± SD, *n* = 6. Paired t-test *p* = 0.52, *p* = 0.13; and one-way ANOVA, Levene’s test *p* > 0.05 followed by Tukey’s HSD test, *p* = 0.678, *p* = 0.871, n.s.—not significant.

Translocation of anionic phospholipids, e.g., phosphatidylserine (PS), to the platelet surface is an important feature of apoptotic and procoagulant platelets. Using fluorochrome-conjugated annexin-V, we tested whether curcumin induces PS surface exposure and/or influences ABT-737-mediated PS exposure. A total of 5 µM of curcumin neither induces PS surface exposure (Figure 1B) nor shows an effect on ABT-737-triggered PS exposure. A total of 50 µM of curcumin induced strong PS exposure, but without any potentiation of 0.1–0.3 µM ABT-737-induced platelet PS surface exposure (Figure 5). These data indicate that curcumin has no additive effects on ABT-737-induced PS surface exposure.

### 2.5. Curcumin Differentially Regulates ABT-737-Induced Platelet Apoptosis

**Figure 6:**
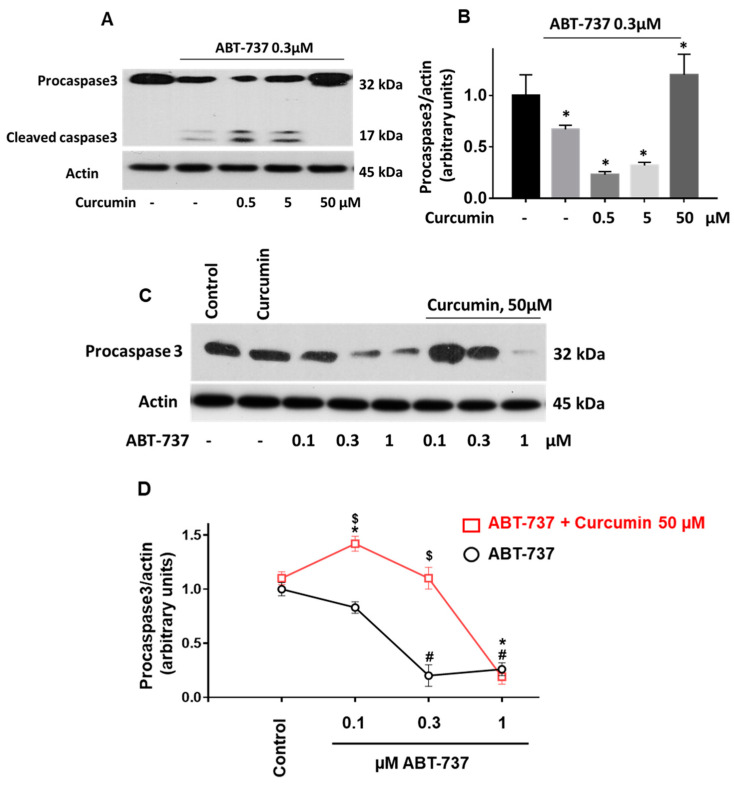
Curcumin at low concentrations stimulated and at high concentration inhibited platelet apoptosis induced by low concentrations of ABT-737. (**A**,**C**) Washed platelets (3 × 10^8^ cells/mL) were incubated with the indicated concentrations of curcumin and ABT-737 for 60 min and analyzed by Western blotting for caspase 3 activation (procaspase 3, cleaved caspase 3). Actin blot served as a loading control. (**B**,**D**) Immunoblots were scanned and the intensity of bands was quantified by the ImageJ program. The intensity of the procaspase 3 signal was normalized to the actin signal. For each sample, this ratio is relatively expressed to the ratio for the control, which is presented as 1. Data are presented as means ± SD. For 6B: One-way ANOVA, Levene’s test *p* > 0.05 followed by Tukey’s HSD test * as appeared in the figure, *p* = 0.017; 0.001; 0.002; 0.005 compared to control, presented as 1. For 6D: One-way ANOVA, Levene’s test *p* > 0.05 followed by Tukey’s HSD test, compared to the corresponding controls, * as appeared in the figure *p* = 0.005; 0.002; # as appeared in the figure *p* = 0.001; 0.001; Paired t-test (“ABT-737 + Curcumin” vs. “ABT-737”) $ as appeared in the figure, *p* = 0.004; 0.002; *n* = 4.

The relationship between autophagy and apoptosis in different cells is well-known but still controversially discussed [46]. In chondrocytes and neuronal cells curcumin induces autophagy and protects these cells from apoptosis [47,48]. Induction of autophagy reduces apoptosis and increases the viability of platelets with immune thrombocytopenia [49]. Therefore, we evaluated whether curcumin modulates ABT-737-induced caspase 3 activation in platelets. First, we tested different curcumin concentrations on caspase 3 activity. Surprisingly, very low curcumin concentrations (0.5–5 µM) significantly potentiated ABT-737 (0.3 µM)-induced caspase 3 activation, whereas a high curcumin concentration (50 µM) inhibited caspase 3 activity (Figure 6A,B).

Next, we compared the effect of 50 µM of curcumin on ABT-737-induced caspase 3 activation in a dose-dependent manner (0.1–1 µM). Curcumin at this concentration significantly prevented 0.1 and 0.3 µM ABT-737-induced procaspase 3 cleavage. However, ABT-737-induced procaspase 3 cleavage induced by a high ABT-737 concentration (1 µM) was not significantly affected by 50 µM of curcumin (Figure 6 C,D).

### 2.6. Curcumin Inhibits Platelet-Dependent Thrombin Generation Mediated by ABT-737

**Figure 7:**
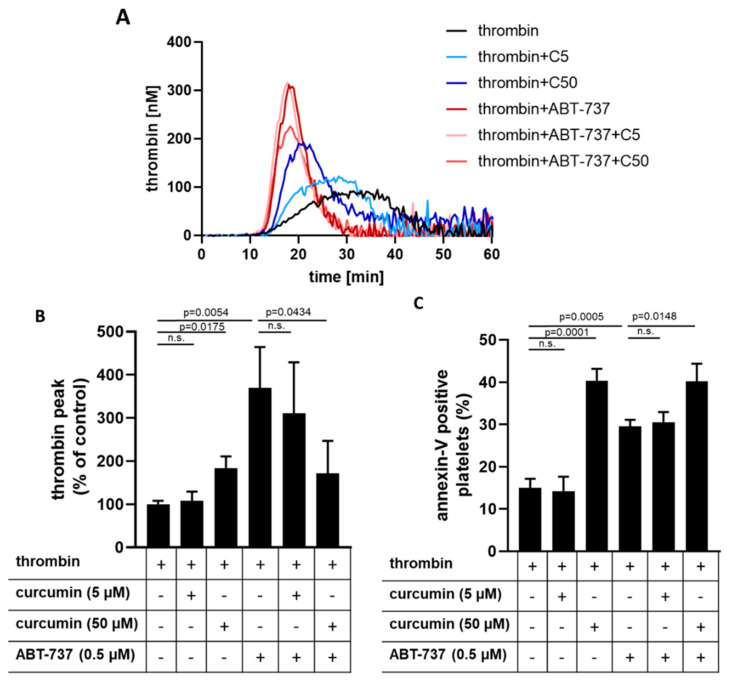
Curcumin at high concentration enhances thrombin-triggered and abolishes ABT-737-mediated thrombin generation on human platelets. Washed platelets (9 × 10^8^ cells/mL) were preincubated with curcumin (5 µM/C5, 50 µM/C50) and/or ABT-737 (0.5 µM) for 60 min, adjusted to 1.5 × 10^8^/mL with autologous platelet-free plasma. Platelet-dependent thrombin generation capacity triggered by α-thrombin (0.1 U/mL) was monitored by calibrated automated thrombography. (**A**) Representative thrombogram curves. (**B**) Quantitative thrombin generation data expressed as thrombin peak in % compared to thrombin control. (**C**) Quantitative data of annexin-V-PE binding to washed human platelets in platelet-free plasma induced by 0.1 U/mL α-thrombin in the absence or presence of curcumin and/or ABT-737, expressed as a percentage of annexin-V-PE positive platelets. Data are presented as means ± SD of 3 separate experiments from 3 different donors (One-way ANOVA, Levene’s test *p* > 0.05 followed by Tukey’s HSD test), n.s. not significant.

In this study, we observed that a high concentration of curcumin (50 µM) induced platelet PS surface exposure on the platelet surface without potentiating ABT-737-mediated PS exposure. On the other hand, curcumin inhibited thrombin-induced platelet αIIbβ3 integrin activation and intracellular Ca^2+^ mobilization after 60 min preincubation. Interestingly, thrombin-induced Ca^2+^ mobilization was completely abolished by ABT-737 (0.5 µM) after 60 min preincubation and not further affected by curcumin (Figure S2). Therefore, we examined whether curcumin affects platelet-dependent thrombin generation triggered by thrombin in the absence or presence of ABT-737. Only 50 µM but not 5 µM curcumin significantly increased thrombin generation/peak in the presence of thrombin (Figure 7). In comparison, the thrombin peak induced by 0.5 µM of ABT-737 in the presence of thrombin was about 2-fold higher. Surprisingly, high curcumin concentration (50 µM) significantly inhibited ABT-737-induced thrombin generation on washed platelets in platelet-free plasma to the level as observed for thrombin plus 50 µM curcumin (Figure 7A,B). For the thrombin generation over time, expressed by the endogenous thrombin potential (etp), also 50 µM of curcumin induced a significant elevation compared to thrombin-treatment alone (965.8 ± 82.24 nM × min vs. 1281 ± 18.65 nM × min, *p* < 0.001), which was further increased by ABT-737 (965.8 ± 82.24 nM × min vs. 1581 ± 100.8 nM × min, *p* < 0.001). Similar to the thrombin peak, the etp induced by ABT-737 was significantly inhibited by 50 µM curcumin (1581 ± 100.8 nM × min vs. 570 ± 53.90 nM × min, *p* < 0.001), too.

Interestingly, the percentage of annexin-V positive platelets induced by 50 µM curcumin plus thrombin was not affected in the presence of ABT-737. These data demonstrate that curcumin at high concentration (50 µM) potentiates thrombin formation on human platelets, but prevents ABT-737-mediated thrombin generation although PS surface exposure was not diminished.

## 3. Discussion

Curcumin alone or in combination with different drugs is widely used for the treatment of many diseases. However, the direct effects of curcumin on platelet function are not fully understood. Applied in vivo, curcumin has a clear antithrombotic effect [31] and this effect, at least partly could be explained by the inhibition of platelet activation induced by different agonists. However, the formation of apoptotic [33,34] or procoagulant platelets also prevents platelet activation [50]. We confirmed previous observations that curcumin at high concentration inhibits thrombin-induced platelet activation [10,12,13], and presented new data that curcumin induces strong platelet PS surface exposure. Curcumin neither activated platelets nor induced caspase 3-mediated apoptosis. Only 50 µM of curcumin slightly reduced platelet viability, suggesting that curcumin-mediated PS surface is directly linked to the formation of procoagulant platelets.

Curcumin is often used in combination with other drugs, including anticancer drugs and the beneficial effects of curcumin, at least partly, are mediated by inhibition of P-gp activity which increases drugs concentration in cells [16,51]. However, for platelets such an increase could be critical, because many anticancer drugs induce platelet apoptosis, or procoagulant platelet formation. On the other hand, clinically used P2Y_12_ receptor inhibitors (e.g., clopidogrel, ticagrelor) are also transported from cells by P-gp [52] and its inhibition might have beneficial effects for the prevention of platelet activation by increasing their intracellular concentration. Until now, it was not known whether curcumin, as in nucleated cells, inhibits P-gp activity in platelets and our results clearly demonstrated it. This information should definitely be considered in clinical applications of curcumin.

Furthermore, curcumin induced autophagy in platelets, expressed by the translocation of LC3I to LC3II as well as by activation of the AMP kinase and inhibition of PKB (Figure 3 and Figure 4). Autophagy in cells is associated with both cell survival and cell death [53,54]. In platelets, autophagy is observed during activation and plays an essential role in thrombosis and hemostasis [55,56]. On the other hand, enhanced autophagy protects against apoptosis in immune thrombocytopenia [49] and oxidative stress in platelets from diabetic patients [57]. In contrast to freshly isolated platelets, autophagy in stored platelets suppresses their activation [58]. In this study, 50 µM of curcumin induced detectable autophagy in platelets. However, the question whether and how activation of autophagy is involved in the changes of platelet functions induced by curcumin is still open and requires further studies in the future. Our results showed for the first time that curcumin induces a procoagulant platelet state (strong PS surface exposure, microparticle formation, decrease of mitochondrial membrane potential). In contrast to cancer cells [18,27], curcumin did not mediate apoptosis of human platelets. However, it dose-dependently modulated caspase 3 activation in response to ABT-737. A low concentration of curcumin potentiated, but a high concentration inhibited ABT-737-induced caspase 3 activation. These results indicate that depending on the concentration curcumin may protect against or inhibit apoptosis of cells, which should be considered for clinical application of curcumin.

In accordance with others [59], we confirmed that ABT-737 potentiates platelet-based thrombin formation in vitro, which could be explained by caspase 3-dependent PS exposure. Curcumin at a high dose also potentiated thrombin formation on platelets, which might be due to the formation of procoagulant platelets. Interestingly, PS surface exposure was not potentiated in the presence of ABT-737 and a high concentration of curcumin when thrombin was absent or present. However, ABT-737-mediated thrombin formation was strongly inhibited in the presence of curcumin at high concentration on thrombin-stimulated platelets. Thus, the inhibitory effect of 50 µM curcumin on ABT-737-induced thrombin generation could not be explained by its modulation of PS exposure. Recently, we showed that the amplification of thrombin generation on thrombin-stimulated platelets is triggered by a fibrin-dependent pathway via CD36, where PS exposure is not essentially required [60]. Therefore, we conclude that the effect of 50 µM curcumin on ABT-737-induced thrombin generation cannot be generally explained by PS exposure, but rather by autophagy. Nevertheless, the identification of the molecular mechanisms of the interplay between curcumin-induced autophagy and modulation of caspase-dependent apoptosis merits future investigation.

In summary, our results demonstrate for the first time that platelet inhibition by curcumin is associated with caspase 3-independent procoagulant platelet formation, which results in strong PS surface exposure, thrombin generation, and induction of autophagy. Curcumin may enhance or protect caspase 3-dependent apoptosis and amplification of thrombin formation on platelets under certain conditions. Therefore, the platelet status should be carefully monitored in patients who are frequently treated with the combination of anticancer drugs and curcumin.

## 4. Materials and Methods

### 4.1. Materials

Curcumin, doxorubicin, LC3 antibody (cat. # L7543), cyclosporine A, and calcein-AM were purchased from Sigma-Aldrich (Munich, Germany). Human α-thrombin was from Roche (Mannheim, Germany). ABT-737 was from Selleckchem (Munich, Germany), actin (cat. # 4970), phospho-AMPK (cat. # 2535), phospho-PKB (cat. # 4060), total PKB (cat. # 4060), total AMPK (cat #2532), and caspase 3 (cat. # 9662) antibodies were obtained from Cell Signaling (Frankfurt, Germany). PAC-1-FITC and annexin-V-PE were from BD Biosciences (Heidelberg, Germany), CD42a-PE antibody, and TMRE dye were from Invitrogen (Waltham, MA, USA). Horseradish peroxidase-conjugated anti-mouse and anti-rabbit IgG antibodies were obtained from Amersham Biosciences Europe GmbH (Freiburg im Breisgau, Germany). Fluo-3 AM was from Life Technologies (Carlsbad, CA, USA). Flow check yellow green (YG) size range calibration kit was from Polyscience Int (Eppelheim, Germany). Thrombin calibrator was purchased from Stago Deutschland GmbH (Düsseldorf, Germany).

### 4.2. Human Platelet Preparation

The study was performed according to the Declaration of Helsinki and the guidelines of the Institute. All participants, healthy volunteers, signed the informed consent before the inclusion in this study. Human platelets were prepared as described previously [61,62]. In brief, citrated whole blood was drawn by venipuncture and collected into Acid Citrate Dextrose (ACD) solution (12 mM citric acid, 15 mM sodium citrate, 25 mM D-glucose) and centrifuged (200× *g*, 10 min, room temperature (RT)) with the preliminary addition of EGTA (2 mM) to generate platelet-rich plasma (PRP). To reduce leukocyte contamination, PRP was resuspended in Citric acid-Glucose-Sodium chloride (CGS) buffer (120 mM NaCl, 12.9 mM trisodium citrate, 10 mM D-glucose, pH 6.5) in a 1:1 ratio and centrifuged (240× *g*, 10 min). After that the supernatant was centrifuged (10 min, 430× *g*), pelleted platelets were washed in CGS buffer, resuspended in HEPES buffer (150 mM NaCl, 10 mM HEPES, 5 mM KCl, 1 mM MgCl_2_, 1mM CaCl_2_, 5 mM d-glucose, pH 7.4) and adjusted to the final concentration of 3 × 10^8^ platelets/mL for Western blot analysis and 1 × 10^8^ platelets/mL for flow cytometry. Then washed platelets rested in the water bath (15 min, 37 °C) and were used in the experiments.

#### Platelet-Free Plasma Preparation

The platelet-poor plasma (PPP) was obtained by centrifugation of citrated whole blood (2000× *g*, 10 min, RT). Platelet-free plasma was prepared by centrifugation of PPP (30,000× *g*, 10 min, RT).

### 4.3. Flow Cytometric Analysis of Platelets

For the analysis of platelet αIIbβ3 integrin activation, PS surface exposure, mitochondrial membrane potential, microparticle formation, and intracellular calcium concentration changes, BD FACSCanto II (BD Biosciences, San Jose, CA, USA) and Navios (BeckmanCoulter, Brea, CA, USA) flow cytometers were used. All the experiments were performed with an analysis of no less than 15,000 events. The data were then anatomized by BD FACSDiva v6.1.3 (BD Biosciences, San Jose, CA, USA) and Cytometry List Mode Data Acquisition & Analysis Software (BeckmanCoulter, Brea, CA, USA), or FlowJo v10.0.7 (FlowJo, LLC, Becton, Dickinson and Company, Franklin Lakes, NJ, USA). The high concentration of curcumin (50 µM) resulted in a substantial increase in platelet autofluorescence detectable in the FL1 channel. Therefore, this signal was subtracted from PAC-1 and calcein-AM fluorescence signals. In contrast, the autofluorescence of curcumin in the FL2 channel was very low and did not interfere with the results of PE-conjugated annexin-V binding to human platelets.

#### 4.3.1. Analysis of Platelet αIIbβ3 Integrin Activation, PS Surface Exposure, and Mitochondrial Membrane Potential

Washed platelets (1 × 10^8^ cells/mL) were incubated with CD42a-PE antibody (1:10, 10 min) and gated by size properties and CD42a positive cells. For the detection of activated αIIbβ3 integrin or surface PS exposure, washed platelets (50 µL) were stained with PAC-1-FITC antibody or annexin-V-PE for 10 min at RT after stimulation with curcumin for indicated concentrations/time. To stop the reaction the platelets were diluted with annexin-V-binding solution (140 mM NaCl, 10 mM HEPES, 2.5 mM CaCl_2_) for annexin-V-PE detection or with PBS for PAC-1-FITC detection and promptly analyzed by flow cytometry. Mitochondrial membrane potential (∆Ψm) was analyzed with TMPE dye. Washed platelets were labelled by TMPE (dilution 1:10) for 10 min at RT after incubation with curcumin for indicated concentrations/time and immediately analyzed by flow cytometry.

#### 4.3.2. Microparticle Formation

Microparticle formation and characterization were performed in the same platelet samples as described previously [63]. Briefly, microparticles were distinguished from platelets according to their size in the forward scatter (FSC)/side scatter (SSC) plot with further marking as CD42a-PE positive in the gate B built according to 0.5–2 µm beads localization. Microparticles were quantified according to CD42a-PE positive events in the gate B. The events less than 0.5 µm were aborted due to the low CD42a fluorescence and high number of unspecific particles of non-platelet origin.

#### 4.3.3. Doxorubicin Accumulation

Doxorubicin, anticancer drug, is a fluorescent P-glycoprotein substrate and is used for screening inhibitors of multidrug resistance [38]. Platelets were stained by doxorubicin (20 µM) for 30 min. Platelets were washed with PBS and resuspended in 0.1% BSA-PBS, then analyzed by flow cytometer with 488 nm excitation/525 nm emission wavelength. Platelets were divided into two gates with different fluorescence intensity. Gate B contained control platelets; gate A contained platelets with increased amount of doxorubicin. Distribution of platelets in each gate was calculated as a percent of all analyzed events (30,000) taken as 100%.

#### 4.3.4. Platelet Ca^2+^-Mobilization

To monitor the intracellular Ca^2+^ concentration changes, washed platelets (3 × 10^8^ platelets/mL) were stained with Fluo-3-AM dye (5 µM, 30 min, 37 °C). Then the cells were monitored for 150 s with an excitation/emission of 488 nm/520 nm in the absence of additional extracellular Ca^2+^. A high concentration of curcumin (50 µM) gave a substantial increase in platelet autofluorescence detectable in the FL1 channel. Therefore, this signal was subtracted from Fluo-3 fluorescence signals prior to and after the addition of thrombin. The ratio of mean fluorescence intensity (MFI) was calculated from treated platelets vs. untreated platelets over time [64].

### 4.4. Western Blot Analysis

Washed platelets (3 × 10^8^ cells/mL) were stimulated with the indicated compounds and lysed in Laemmli sample buffer. Proteins were separated by SDS-PAGE, transferred to nitrocellulose membranes, and incubated with appropriate primary antibodies (overnight, 4 °C). Conjugated with horseradish peroxidase goat anti-rabbit or anti-mouse IgG were used as secondary antibodies with the following enhanced chemiluminescent (ECL) detection (GE Healthcare, Chicago, IL, USA) for signal visualization. Actin blots were used as a loading control for quantitation of Western blot data. Thrombin (0.01 U/mL, 1 min) was used as a positive control for PKB and AMPK phosphorylation. For the densitometrical analysis of the immunoblots ImageJ software (National Institutes of Health, Bethesda, MD, USA and Laboratory for Optical and Computational Instrumentation, Madison, WI, USA) for uncalibrated optical density was used.

### 4.5. Calibrated Automated Thrombography

Washed platelets (9 × 10^8^ platelets/mL) were preincubated with ABT-737 (0.5 µM) and/or curcumin (5 µM, 50 µM) for 60 min at 37 °C, then adjusted to 1.5 × 10^8^ cells/mL with autologous platelet-free plasma. Platelet-dependent thrombin generation capacity triggered by α-thrombin (0.1 U/mL) was monitored by calibrated automated thrombography as previously described [65]. Thrombinoscope analysis software (V5.0, Diagnostica Stago, Asnieres sur Seine Cedex, France) was used to calculate thrombograms and thrombin generation parameters.

### 4.6. Data Analysis

For data analysis IBM SPSS Statistics v.26 (IBM Corporation, Armonk, NY, USA) or GraphPad Prism v.9 (GraphPad Software Inc., San Diego, CA, USA) were applied. According to Shapiro–Wilk’s test the data were normally distributed, therefore for group comparisons one-way ANOVA was used. According to Levene’s test, Tukey HSD, or Tamhane’s T2 post-hoc analysis were used where appropriate. One-sample or paired t-test were used where applicable. Each dataset represents not less than three different experiments on the material taken from not less than three different healthy volunteers. Data are presented as the Mean ± SD, *p* < 0.05 was considered to be statistically significant.

## Data Availability

Not applicable.

## Supplementary Materials

The following are available online at https://www.mdpi.com/article/10.3390/ijms22105405/s1. References [66, 67, 68] are cited in the supplementary materials.
