# Supplementary material for: Curcumin at Low Doses Potentiates and at High Doses Inhibits ABT-737-Induced Platelet Apoptosis
Source: Int J Mol Sci. 2021 May 20;22(10):5405. doi: 10.3390/ijms22105405 (PMC8161296; doi:10.3390/ijms22105405)
Supplement: Supplementary file 1 [file ijms-22-05405-s001.zip › ijms-1205717-supplementary.pdf]

Supplementary Information

Curcumin at low doses potentiates, and at high doses inhibits ABT-737-induced platelet apoptosis

Natalia Rukoyatkina[1], Valentina Shpakova[1], Julia Sudnitsyna[1,2], Michael Panteleev[2], Stephanie Makhoul[3], Stepan Gambaryan[1,3], Kerstin Jurk[3*]

Method for analysis of platelet aggregation.

Platelet aggregation was analysed by low angle light scattering (laser diffraction) method as described (Mindukshev et al, 2012; Reiss et al, 2015). Briefly, PRP was diluted in 1 ml of modified HEPES buffer (pH 7.4, osmolarity 302 mOsm, containing 140 mM NaCl, 10 mM HEPES, 10 mM $NaHCO_3$, 2 mM KCl, 1 mM $MgCl_2$, 2 mM $CaCl_2$, 5.5 mM D-glucose) to a concentration of $1x10^7$ platelets/ml. After 2 minutes of establishing a constant basal signal, ADP (300 nM, used as a positive control) or curcumin (50 µM) were added to the platelet suspension, and the recordings were performed for additional 5 min. Platelet aggregation was recorded as increase of light scattering intensity at 1 degree (LSI 1°).

Supplementary Figures

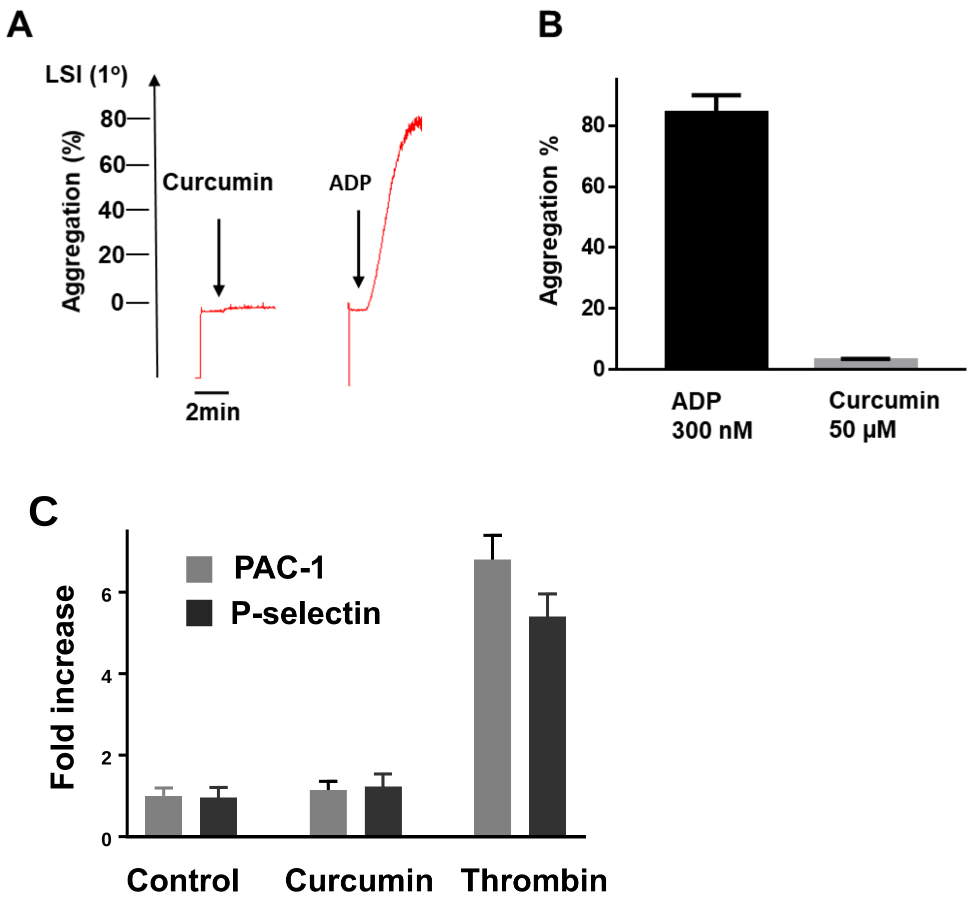

Figure S1. Curcumin itself does not affect platelet activation. (**A**) Representative curves of platelet aggregation in response to curcumin and ADP, respectively. (**B**) Quantification of aggregation data from four independent experiments. Data are presented as means ± SD, n = 4. (**C**) Washed platelets ($1x10^8$/ ml) were incubated with curcumin (50 µM, 10 min) or thrombin (0.01 U/ml, 2 min) as a positive control. PAC-1, or P-selectin antibodies were added for additional 5 min, then platelets were fixed with 2% (final concentration) formaldehyde, washed by PBS and analyzed by flow cytometry. Data are presented as fold increase of control sample set as 1 and presented as means ± SD, n = 4.

A

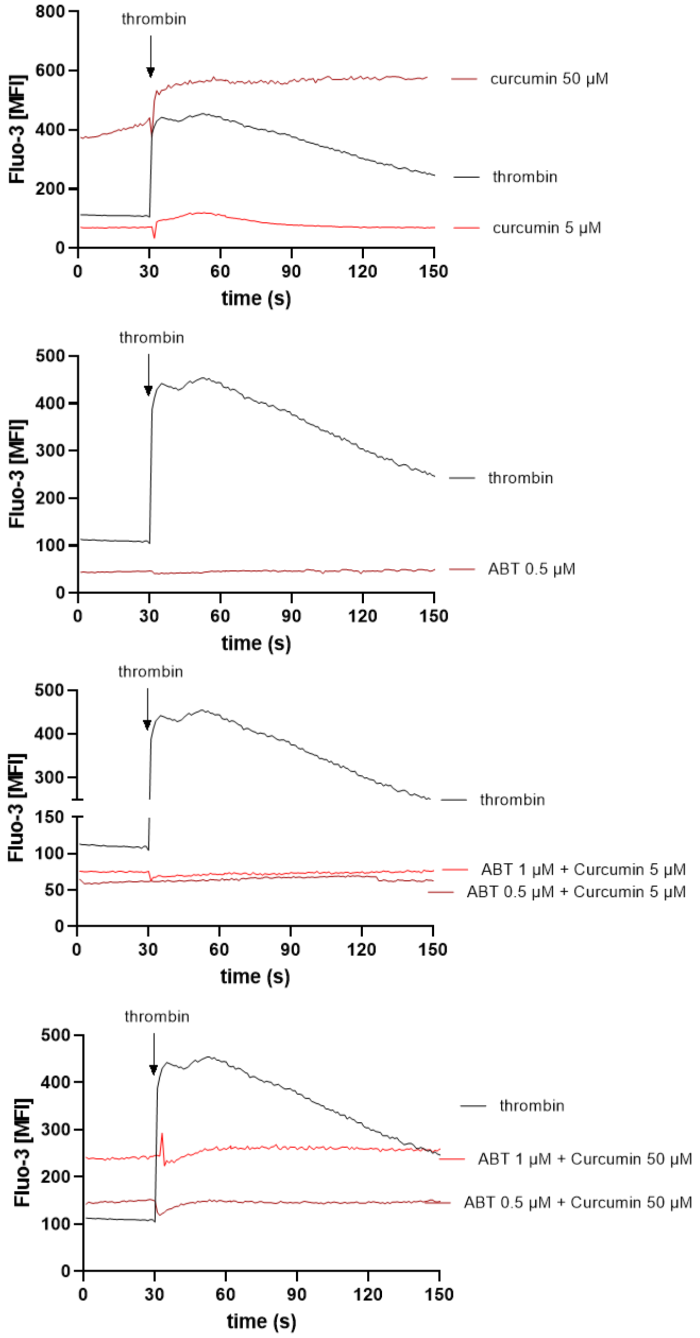
thrombin
Fluo-3 [MFI]
time (s)
curcumin 50 µM
thrombin
curcumin 5 µM
ABT 0.5 µM
ABT 1 µM + Curcumin 5 µM
ABT 0.5 µM + Curcumin 5 µM
ABT 1 µM + Curcumin 50 µM
ABT 0.5 µM + Curcumin 50 µM

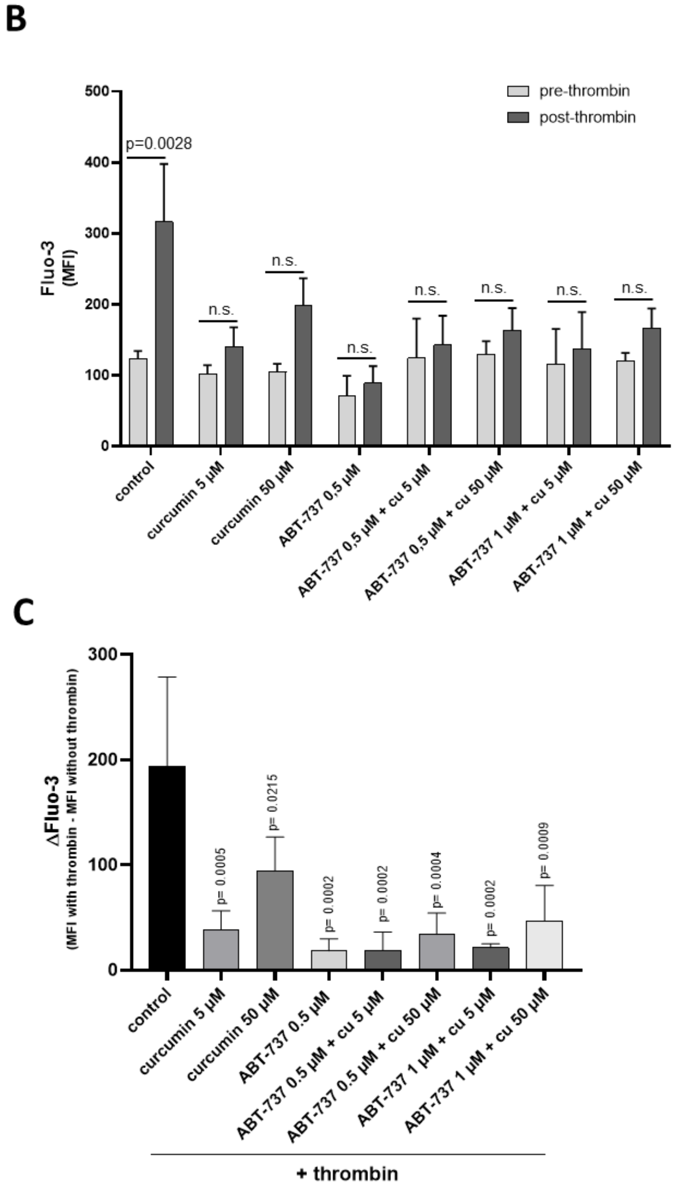

Figure S2. Curcumin inhibits thrombin-induced Ca^{2+}-mobilization. Washed platelets (3 x 10^8 platelets/ml) were loaded with 5 µM Fluo-3-AM for 30 min at 37°C. Platelets were stimulated with 0.05 U/ml thrombin in the absence or presence of curcumin and/or ABT-737 (preincubation for 60 min at 37°C). Fluo-3-AM fluorescence was monitored for 150 s in the absence of additional extracellular Ca^{2+} by flow cytometry. (**A**) Representative traces of intracellular Ca^{2+} mobilization. (**B**) Quantification of Fluo-3-AM mean fluorescence intensity (MFI) before (pre-thrombin) and after addition of thrombin (post-thrombin); curcumin autofluorescence was subtracted from 50 µM curcumin samples (one-way ANOVA, Leven's test p>0.05 followed by Tukey's HSD test, n=3). (**C**) Quantification of delta Fluo-3 mean fluorescence intensity (MFI) calculated by subtraction of maximum MFI in response to thrombin with maximum MFI under basal conditions for each condition as shown in (B) (one-way ANOVA, Leven's test p>0.05 followed by Tukey's HSD test, n=3).

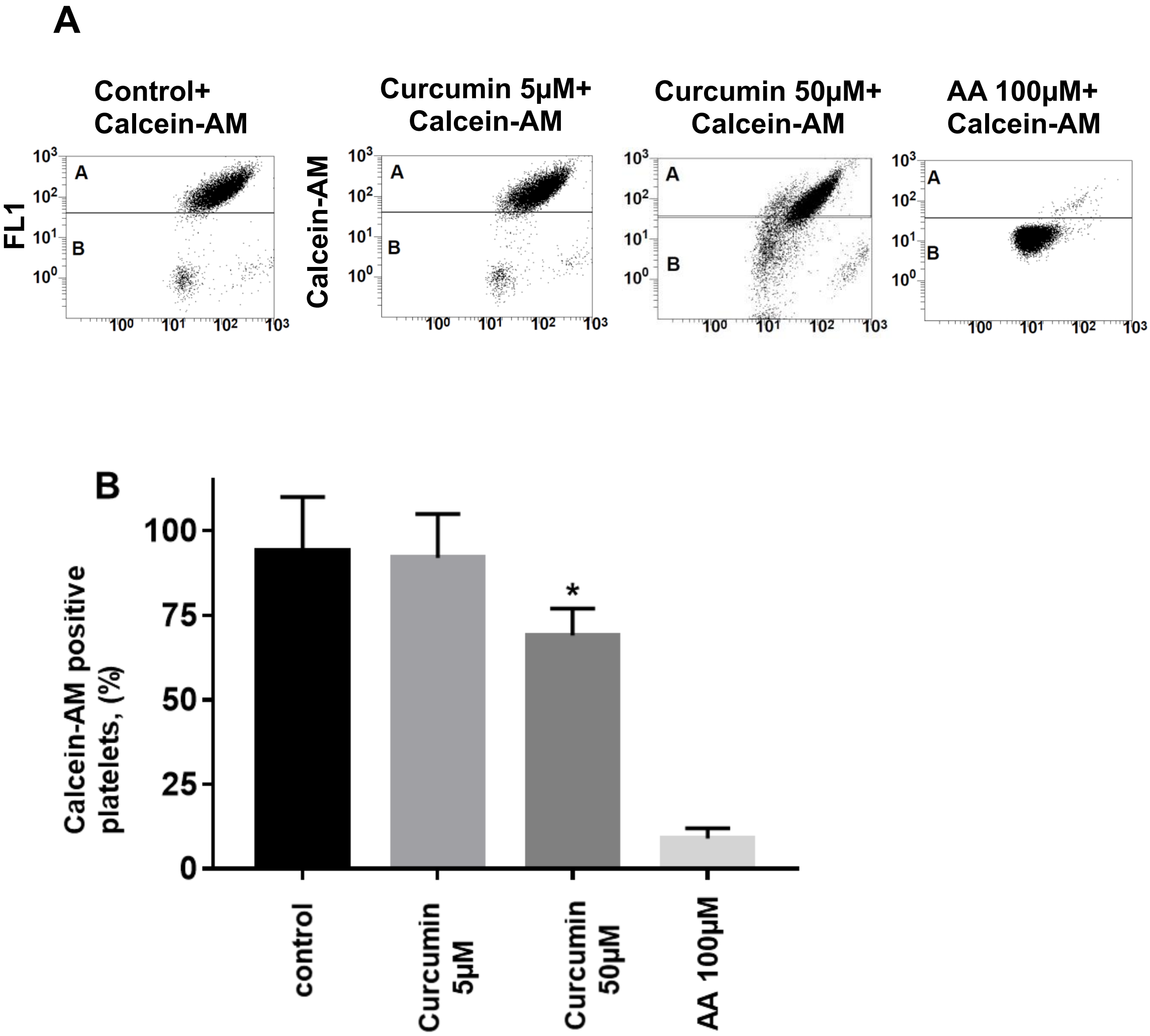

Figure S3. Effect of curcumin on platelet viability. Washed platelets (1x10^8 /ml) were preincubated with Calcein-AM (0.2 µM, 30 min). Indicated concentrations of curcumin were added for additional 60 min and analyzed by flow cytometry for Calcein-AM positive platelets. Arachidonic acid (AA, 100 µM), which decreases esterase activity more than 95% (Rukoyatkina et al, 2018) was used as positive control. (**A**) Representative dot plots, (**B**) quantification of four independent experiments (autofluorescence of curcumin was subtracted from 50 µM curcumin samples). Data are presented as means ± SD (One-way ANOVA, Leven's test P>0.05 followed by Tukey's HSD test, *, P = 0.049 compared to control, n = 5).

References:

66. Mindukshev I, Gambaryan S, Kehrer L, Schuetz C, Kobsar A, Rukoyatkina N, Nikolaev VO, Krivchenko A, Watson SP, Walter U, Geiger J. Low angle light scattering analysis: a novel quantitative method for functional characterization of human and murine platelet receptors. *Clin Chem Lab Med*. **50**, 1253 – 1262 (2012).

67. Reiss C, Mindukshev I, Bischoff V, Subramanian H, Kehrer L, Friebe A, Stasch JP, Gambaryan S, Walter U. The sGC stimulator riociguat inhibits platelet function in washed platelets but not in whole blood. *Br J Pharmacol*. **172**, 5199-210 (2015).

68. Rukoyatkina N, Shpakova V, Panteleev M, Kharazova A, Gambaryan S, Geiger J. Multifaceted effects of arachidonic acid and interaction with cyclic nucleotides in human platelets. *Thromb Res*. **171**, 22-30 (2018).
